# Cardiac Conduction Disorders in Melanoma Patients Treated with CTLA-4-Containing Versus PD-1-Only Immune Checkpoint Inhibitor Therapy: A Propensity Score-Matched Real-World Analysis

**DOI:** 10.3390/cancers18162608

**Published:** 2026-08-13

**Authors:** Ali Awad, Joe Khodeir, Qusai AlQudah, Nur Saleh, Mariam Chalhoub, M. Chadi Alraies

**Affiliations:** 1 Department of Internal Medicine, Detroit Medical Center, Wayne State University, Detroit, MI 48201, USA; salehnur101@gmail.com; 2 Division of Dermatology, Faculty of Medicine and Medical Sciences, University of Balamand, Tripoli P.O. Box 100, Lebanon; joe.j.khodeir@fty.balamand.edu.lb; 3 Department of Medicine, University of Central Florida College of Medicine, Orlando, FL 32827, USA; ana.qu.sai@hotmail.com; 4 Department of Medicine, University Hospitals Cleveland Medical Center, Case Western Reserve University, Cleveland, OH 44106, USA; mariam.chalhoub@uhhospitals.org; 5 Department of Cardiovascular Medicine, Detroit Medical Center, Wayne State University, Detroit, MI 48201, USA; alraies@hotmail.com

**Keywords:** melanoma, immune checkpoint inhibitors, ipilimumab, CTLA-4, PD-1, cardiotoxicity, conduction disorders, atrioventricular block, propensity score matching, TriNetX

## Abstract

**Background**: Immune checkpoint inhibitors (ICIs) have transformed the treatment of advanced melanoma, but the comparative cardiac safety of cytotoxic T-lymphocyte-associated antigen 4 (CTLA-4)-containing regimens versus programmed cell death protein 1 (PD-1)-only regimens in real-world populations remains poorly characterized. We compared cardiac outcomes between melanoma patients exposed to CTLA-4 blockade (ipilimumab) and those treated with anti-PD-1 therapy alone (nivolumab or pembrolizumab) using a large multicenter electronic health record database. **Methods**: This retrospective cohort study used de-identified data from the TriNetX Research Network (111 US healthcare organizations). Adults with melanoma (ICD-10-CM C43) who received ipilimumab (CTLA-4 exposed) were compared with those who received nivolumab or pembrolizumab without ipilimumab (anti–PD-1 only); both groups were therefore treated with immune checkpoint inhibitors, isolating the effect of CTLA-4 exposure. Propensity score matching (1:1) balanced demographics, cardiovascular comorbidities, and baseline antiarrhythmic use. The primary outcome was a cardiac conduction disorder composite; outcomes were assessed at 1 and 3 years. **Results**: Of 7313 CTLA-4-exposed and 11,481 anti-PD-1-only patients, 6841 matched pairs were analyzed (all standardized mean differences <0.03). CTLA-4 exposure was associated with a significantly higher incidence of cardiac conduction disorders that was already present at 1 year (3.8% vs. 2.1%; RR 1.82; 95% CI 1.48–2.24; *p* < 0.001) and persisted at 3 years (5.1% vs. 3.7%; RR 1.36; 95% CI 1.16–1.60; *p* < 0.001). Atrioventricular block was higher at both 1 year (RR 2.10; 95% CI 1.59–2.78) and 3 years (RR 1.49; 95% CI 1.20–1.85), and complete heart block was markedly increased at 3 years (0.5% vs. 0.2%; RR 3.28; 95% CI 1.67–6.43; *p* < 0.001). Heart failure was modestly higher with CTLA-4 exposure at both timepoints (1-year RR 1.35; 3-year RR 1.17). **Conclusions**: Among melanoma patients treated with immune checkpoint inhibitors, CTLA-4-containing therapy is associated with a higher burden of cardiac conduction disorders, including a roughly three-fold excess of complete heart block, that is evident within the first year and sustained thereafter. Because the CTLA-4-exposed cohort comprised both ipilimumab monotherapy and nivolumab plus ipilimumab and the subgroup analyses localized the excess risk to the combination regimen, this association reflects CTLA-4-containing (predominantly combination) therapy rather than ipilimumab monotherapy in isolation. These findings support electrocardiographic surveillance beginning during, not only after, treatment for patients receiving CTLA-4-containing immunotherapy.

## Simple Summary

Immune therapies have greatly improved survival for people with advanced melanoma, the most serious form of skin cancer, but they can occasionally affect the heart. Using a large database of anonymized records from 111 United States health systems, we compared melanoma patients treated with ipilimumab, a drug that blocks the immune checkpoint CTLA-4, against patients treated only with drugs that block a different checkpoint, PD-1 (nivolumab or pembrolizumab). After matching the two groups so that they were similar, we found that patients who received the CTLA-4 drug had a higher risk of disorders of the heart’s electrical conduction system, including an almost three-fold higher risk of complete heart block. This risk appeared within the first year and persisted for at least three years. These patients may benefit from regular heart-rhythm monitoring during and after treatment.

## 1. Introduction

Melanoma accounts for approximately 1% of skin cancer diagnoses, yet it is responsible for the majority of skin cancer-related deaths worldwide [1]. Over the past decade, outcomes for patients with advanced melanoma have been fundamentally transformed by two distinct systemic therapy classes: BRAF/MEK-targeted agents and immune checkpoint inhibitors (ICIs) [1]. These advances have shifted the disease from a historically fatal condition with a median overall survival of less than 12 months to one in which durable long-term remission is achievable for a substantial proportion of patients [2,3].

Approximately 40–50% of cutaneous melanomas harbor activating BRAF V600 mutations [4], enabling BRAF/MEK-targeted regimens that improve response rates and overall survival in BRAF-mutant disease [4,5,6,7]. Immune checkpoint inhibitors targeting the programmed cell death protein 1 (PD-1) pathway (pembrolizumab, nivolumab) and the cytotoxic T-lymphocyte-associated antigen 4 (CTLA-4) pathway (ipilimumab) have redefined treatment expectations across all melanoma genotypes, with 5-year survival exceeding 50% with combination nivolumab plus ipilimumab in pivotal trials [5,8,9,10,11,12].

Despite their efficacy, immune checkpoint inhibitors carry distinct cardiovascular toxicity profiles. They may trigger immune-mediated cardiotoxicity, including myocarditis, pericarditis, and conduction system disease [13,14,15]. ICI-associated myocarditis, though occurring in approximately 1% of patients, carries a case fatality rate exceeding 40%, making early recognition critical [13,16]. This toxicity is not uniform across regimens, and CTLA-4 blockade in particular has been implicated in more frequent and more severe immune-related cardiac events [17,18].

Cardiac conduction system involvement has emerged as a particularly underrecognized complication of checkpoint inhibition, arising from T-cell-mediated inflammation of the conduction axis and potentially progressing to atrioventricular block or malignant arrhythmia [19]. Pharmacovigilance data suggest conduction disorders represent approximately 20% of all ICI-related cardiac events, with a predilection for CTLA-4-containing regimens [17,18]. Yet real-world data directly comparing the cardiac risk of CTLA-4-containing versus PD-1-only immunotherapy remain limited, with most evidence derived from clinical trials with cardiac exclusion criteria or single-institution case series [13,17,18].

In this large retrospective analysis using the TriNetX Research Network, we compared cardiac conduction disorders, heart failure, and ventricular tachycardia between propensity score-matched melanoma patients exposed to CTLA-4 blockade (ipilimumab) and those treated with anti-PD-1 therapy alone (nivolumab or pembrolizumab) at 1 and 3 years. Because both groups received immune checkpoint inhibitors, this design isolates the incremental cardiac risk attributable to CTLA-4 exposure while holding disease severity and surveillance intensity approximately constant.

## 2. Methods

### 2.1. Study Population

This retrospective cohort study utilized de-identified electronic health record data from the TriNetX Research Network, a global federated health research database comprising electronic medical records from 111 participating healthcare organizations across the United States. Adult patients (≥18 years of age) with a diagnosis of malignant melanoma of skin (ICD-10-CM C43) were identified, with data extraction performed on 15 March 2026. Patients whose index event occurred more than 20 years before data extraction were excluded, consistent with TriNetX analytic standards.

Two mutually exclusive cohorts were defined among melanoma patients treated with immune checkpoint inhibitors. The CTLA-4-exposed cohort included patients who received ipilimumab (RxNorm 1094833) at any time, capturing both ipilimumab monotherapy and nivolumab plus ipilimumab combination therapy. The anti-PD-1-only cohort included patients who received nivolumab (1597876) or pembrolizumab (1547545) with no exposure to ipilimumab at any time, ensuring mutually exclusive groups. Both cohorts required a melanoma diagnosis (ICD-10-CM C43) and age ≥18 years; BRAF/MEK-targeted agents were not used to define either cohort.

### 2.2. Data Collection and Outcomes

Demographic characteristics, diagnoses, procedures, and encounter data were extracted using the TriNetX analytics platform and standardized across institutions using ICD-10-CM, CPT, and HL7 visit types. The index event was the first recorded administration of ipilimumab for the CTLA-4-exposed cohort and of nivolumab or pembrolizumab for the anti–PD-1–only cohort. Clinical outcomes were assessed over two follow-up intervals, 1 year and 3 years, beginning one day after the index event. Before matching, the CTLA-4-exposed cohort comprised 7313 patients and the anti-PD-1-only cohort comprised 11,481 patients; after 1:1 propensity score matching, 6841 patients remained in each cohort, with standardized mean differences <0.03 across all baseline covariates, indicating excellent balance.

### 2.3. Outcomes

The primary outcome was a cardiac conduction disorder composite, defined as any diagnosis of atrioventricular and left bundle-branch block (ICD-10-CM I44), complete atrioventricular block (I44.2), or other conduction disorders (I45). Secondary cardiac outcomes included atrioventricular block (I44), other conduction disorders (I45), first-degree atrioventricular block (I44.0), second-degree atrioventricular block (I44.1), complete atrioventricular block (I44.2), ventricular tachycardia (I47.2), and heart failure (I50). A composite conduction endpoint was used to increase statistical power given the rarity of individual conduction abnormalities. For each outcome, patients with a prior diagnosis of that outcome were excluded from the corresponding risk set, resulting in variable denominators across outcomes. The TriNetX platform normalizes diagnosis codes across contributing sites by mapping ICD-9 to ICD-10 within its standard data model, ensuring consistent outcome ascertainment regardless of the coding system used by individual organizations.

### 2.4. Statistical Analysis

Propensity score matching was performed using a 1:1 nearest-neighbor algorithm with a caliper width of 0.1 standard deviations of the logit of the propensity score. Matching variables included age, sex, race, hypertension, diabetes mellitus, hyperlipidemia, chronic kidney disease, coronary artery disease, heart failure, atrial fibrillation or flutter, obesity, and cerebrovascular disease, as well as baseline antiarrhythmic medication use. Balance between cohorts was evaluated using standardized mean differences (SMDs), with values <0.03 achieved across all covariates. Risk ratios (RRs) with 95% confidence intervals were generated for each outcome using the TriNetX analytics platform, and *p*-values were derived from the associated risk-difference test, implemented in the TriNetX platform as a two-sided Z-test for the difference in proportions (normal approximation). For the rarest outcomes, in which absolute event counts were small, this normal approximation is less robust, and the corresponding confidence intervals and *p*-values should be interpreted with additional caution. All analyses were conducted within the TriNetX platform using real-time statistical computation. A two-sided *p*-value < 0.05 was considered statistically significant. The cardiac conduction disorder composite was the single prespecified primary endpoint; the individual conduction subtypes, ventricular tachycardia, heart failure, and the agent-level subgroup comparisons were prespecified but secondary and exploratory. Given the number of outcomes and subgroups examined (more than twenty statistical tests), no adjustment for multiple comparisons was applied, and the secondary and subgroup results should be regarded as hypothesis-generating; borderline findings near the 0.05 threshold warrant particularly cautious interpretation. Missing data were handled using the TriNetX default analytic framework, which performs complete-case analysis for each outcome without imputation. Because this was a retrospective analysis of all eligible matched patients rather than a prospectively recruited study, no a priori target sample size was set; statistical power was instead assessed after matching. For the primary composite outcome, with a baseline event rate of approximately 2% in the anti–PD-1 arm, the 6841 matched pairs provided roughly 80% power to detect a risk ratio of 1.37 and greater than 99% power for the observed effect (risk ratio 1.82), at a two-sided alpha of 0.05. The agent-level subgroup analyses (3924 to 4937 matched pairs) retained approximately 80% power to detect risk ratios of 1.35 to 1.46; the rarer conduction subtypes, such as second-degree block, were underpowered, and their non-significant results should be interpreted as inconclusive rather than negative.

### 2.5. ICI Agent Subgroup Analyses

To characterize agent-specific cardiac risk within the ICI cohort, three prespecified pairwise subgroup analyses were performed. The comparisons were: (1) nivolumab plus ipilimumab versus pembrolizumab monotherapy; (2) nivolumab monotherapy versus nivolumab plus ipilimumab; and (3) nivolumab monotherapy versus pembrolizumab monotherapy. Mutually exclusive subcohorts were defined as follows: the nivolumab plus ipilimumab group required documented receipt of both agents with no pembrolizumab exposure; the pembrolizumab monotherapy group required pembrolizumab receipt with no nivolumab or ipilimumab exposure; and the nivolumab monotherapy group required nivolumab receipt with no concurrent ipilimumab or pembrolizumab exposure. Each pairwise comparison underwent 1:1 propensity score matching using the same covariates as the primary analysis. Outcomes were assessed over a 1-year follow-up interval from the index event.

### 2.6. Ethical Considerations

Because this study used de-identified aggregate data obtained from the TriNetX Research Network, no institutional review board approval or patient consent was required, in accordance with 45 CFR §46 and institutional policies on exempt research.

## 3. Results

### 3.1. Baseline Characteristics

**Figure 1:**
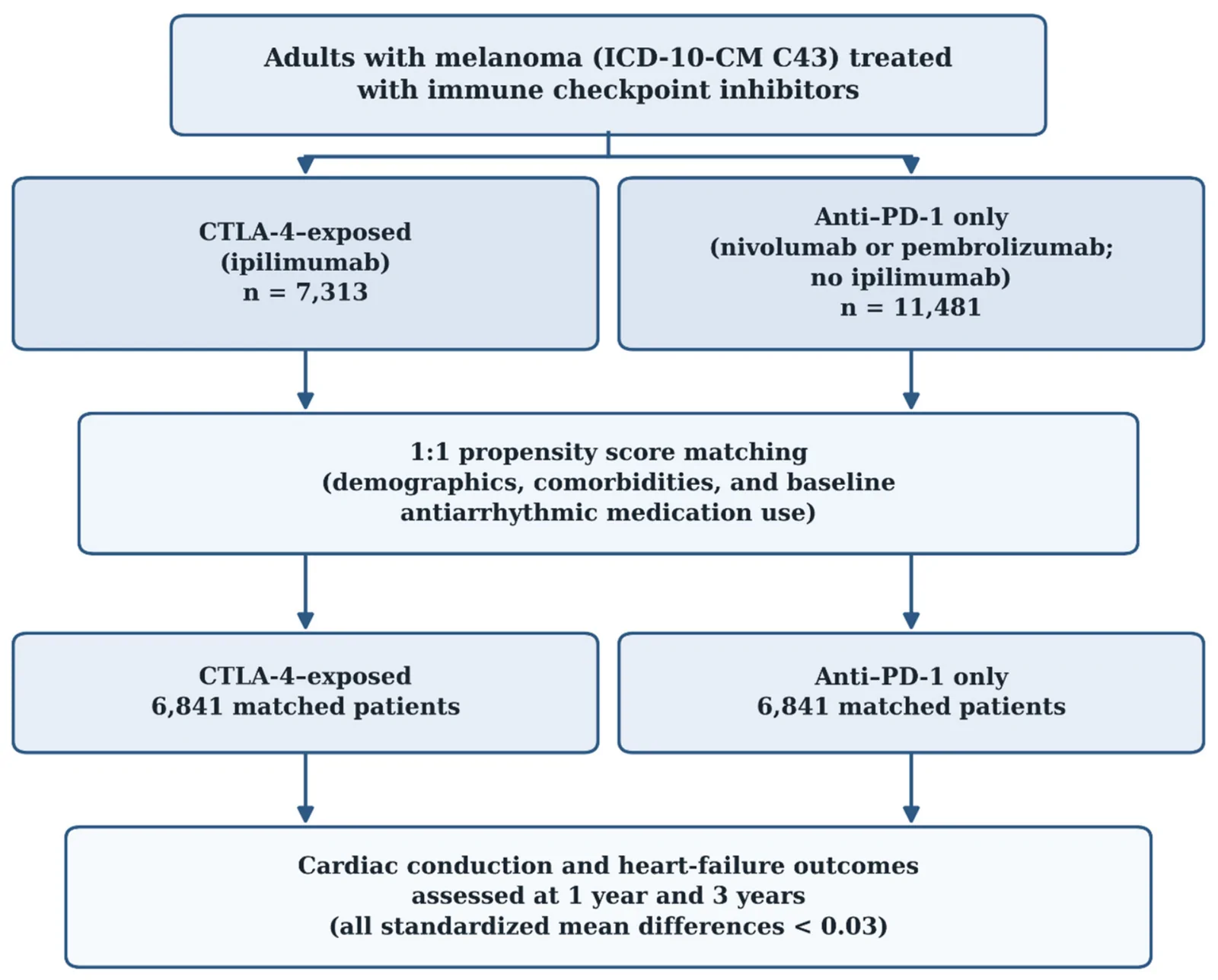
Study Design and Cohort Selection. Flow diagram illustrating patient selection, inclusion and exclusion criteria, and propensity score matching for the CTLA-4-exposed (ipilimumab) versus anti-PD-1-only (nivolumab or pembrolizumab) cohorts in melanoma.

From the TriNetX Research Network, 7313 melanoma patients exposed to CTLA-4 blockade (ipilimumab) and 11,481 treated with anti-PD-1 therapy alone (nivolumab or pembrolizumab) were identified. After 1:1 propensity score matching, 6841 matched pairs were analyzed (Figure 1), with standardized mean differences <0.03 across all baseline covariates, indicating excellent balance (Table 1). Before matching, the CTLA-4-exposed cohort was younger (mean age at index 61.8 ± 14.0 vs. 66.8 ± 14.2 years; SMD 0.353) and had fewer cardiovascular comorbidities, consistent with selection of younger, fitter patients for CTLA-4-containing regimens; these differences were fully resolved after matching (Supplemental Table S2). To our knowledge, this is among the largest real-world analyses to date isolating the cardiac conduction risk attributable to CTLA-4 exposure within immune checkpoint inhibitor-treated melanoma patients.

### 3.2. Clinical Outcomes

Outcomes were assessed over two cumulative follow-up intervals from the index date of therapy initiation: 1 year and 3 years.

#### 3.2.1. One-Year Outcomes

At 1 year, CTLA-4 exposure was associated with a significantly higher incidence of cardiac conduction disorders than anti-PD-1 therapy alone (3.8% vs. 2.1%; RR 1.82; 95% CI 1.48–2.24; *p* < 0.001). This early signal was driven by both atrioventricular block (2.3% vs. 1.1%; RR 2.10; 95% CI 1.59–2.78; *p* < 0.001) and other conduction disorders (2.2% vs. 1.4%; RR 1.53; 95% CI 1.18–1.98; *p* = 0.001). Among atrioventricular block subtypes, first-degree block was significantly more frequent with CTLA-4 exposure (0.8% vs. 0.5%; RR 1.66; 95% CI 1.07–2.57; *p* = 0.022), whereas second-degree block did not differ (0.2% vs. 0.2%; RR 1.25; *p* = 0.562). Heart failure was also significantly more frequent with CTLA-4 exposure (4.0% vs. 2.9%; RR 1.35; 95% CI 1.12–1.62; *p* = 0.001). Ventricular tachycardia showed a borderline trend (1.0% vs. 0.7%; RR 1.42; 95% CI 0.98–2.05; *p* = 0.061) (Table 2).

#### 3.2.2. Three-Year Outcomes

At 3 years, the excess conduction risk with CTLA-4 exposure persisted. The composite conduction disorder rate remained significantly higher (5.1% vs. 3.7%; RR 1.36; 95% CI 1.16–1.60; *p* < 0.001), as did atrioventricular block (3.0% vs. 2.0%; RR 1.49; 95% CI 1.20–1.85; *p* < 0.001) and other conduction disorders (3.0% vs. 2.4%; RR 1.26; 95% CI 1.02–1.54; *p* = 0.029). A graded relationship by block severity was evident: first-degree (RR 1.11; *p* = 0.548) and second-degree (RR 1.47; *p* = 0.248) atrioventricular block did not differ significantly, whereas complete heart block, the most clinically consequential lesion, was markedly more frequent with CTLA-4 exposure (0.5% vs. 0.2%; RR 3.28; 95% CI 1.67–6.43; *p* < 0.001). Heart failure remained modestly higher (5.7% vs. 4.9%; RR 1.17; 95% CI 1.01–1.35; *p* = 0.038), and ventricular tachycardia did not differ significantly (1.4% vs. 1.2%; RR 1.13; 95% CI 0.85–1.52; *p* = 0.401). Consistent findings were observed before matching (Supplemental Table S1). All outcomes are summarized in Table 2.

**Figure 2:**
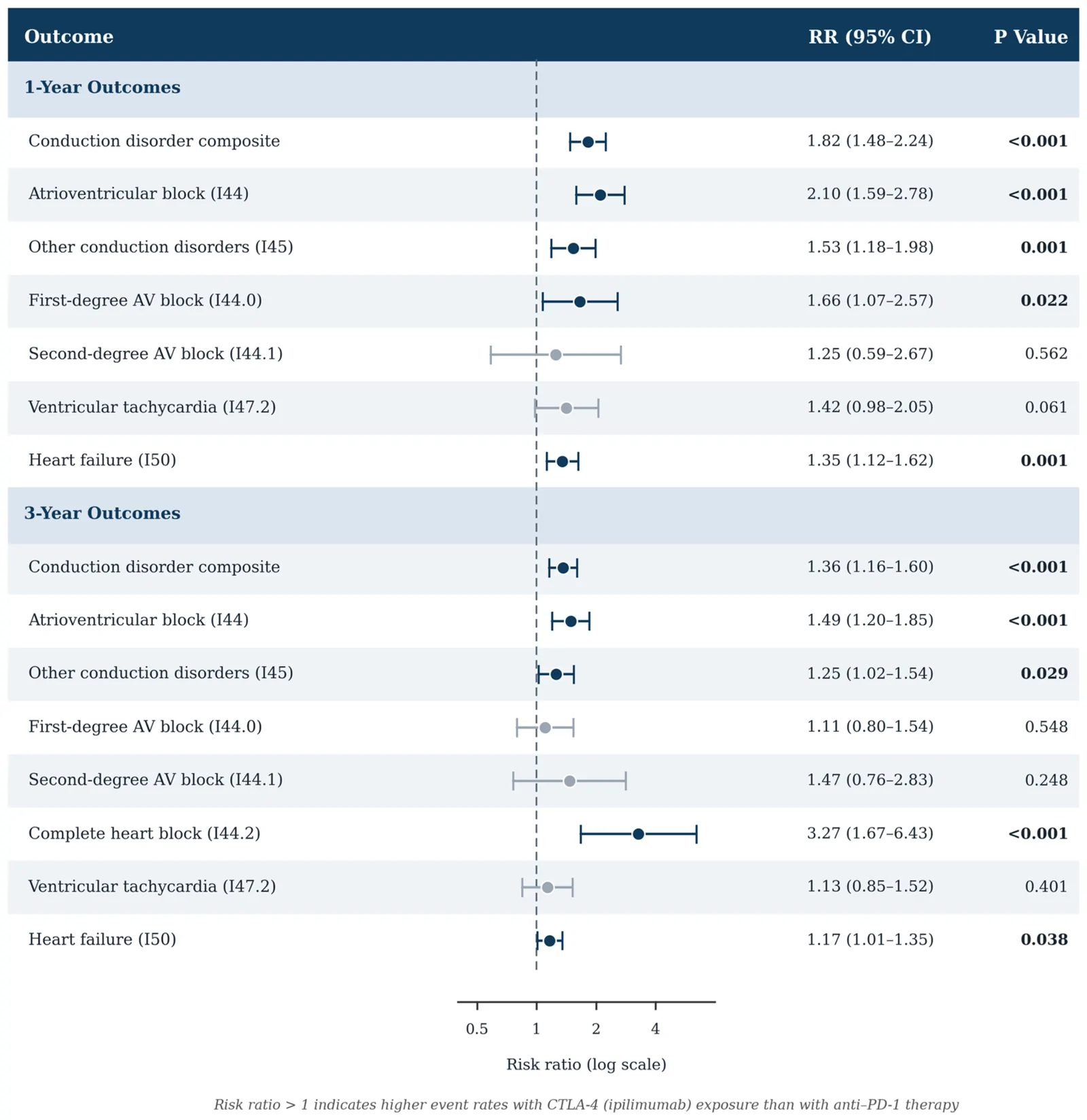
Forest Plot of the Main Analysis. Risk ratio (95% CI) and *p* value for each cardiac conduction and heart-failure outcome at 1 year and 3 years, comparing CTLA-4-exposed (ipilimumab) versus anti-PD-1-only (nivolumab or pembrolizumab) melanoma patients in the propensity score–matched cohort. The dashed vertical line marks the null value (RR = 1.0); navy markers denote statistically significant associations (95% CI excluding 1.0) and grey markers non-significant associations. Risk ratios >1.0 indicate higher event rates with CTLA-4 exposure than with anti-PD-1 therapy.

The forest plot of risk ratios across both timepoints illustrates the early and sustained excess of conduction outcomes with CTLA-4 exposure, including the marked excess of complete heart block by 3 years (Figure 2).

### 3.3. ICI Agent Subgroup Analyses

**Figure 3:**
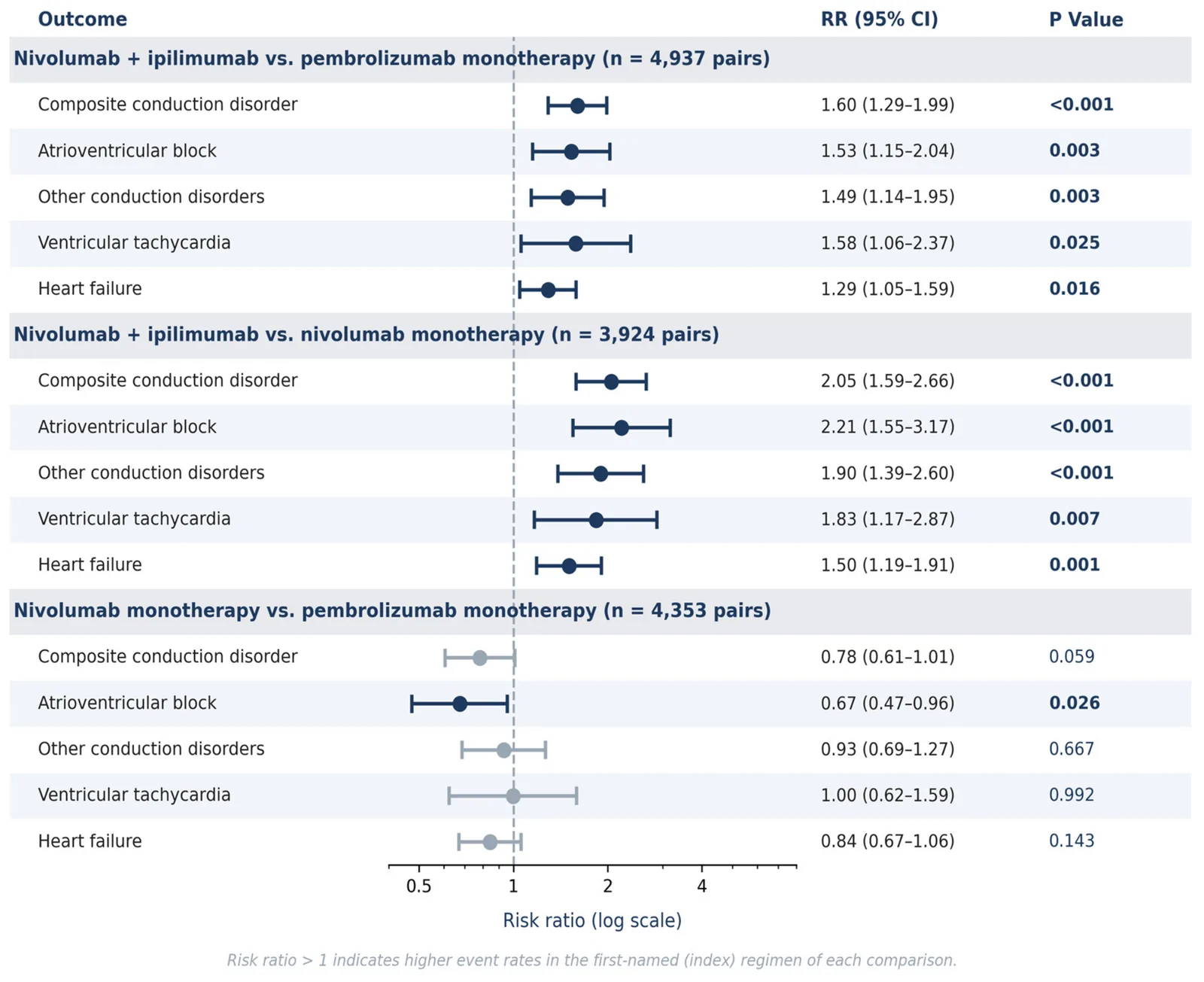
Forest Plot of the Subgroup Analyses. Risk ratio (95% CI) and *p* value for the composite cardiac conduction disorder, atrioventricular block, and heart failure across the three prespecified within-ICI subgroup comparisons at 1 year. Navy markers denote statistically significant associations (95% CI excluding 1.0) and grey markers non-significant associations; risk ratios >1.0 indicate higher event rates in the first-named regimen of each comparison.

Three pairwise subgroup analyses were performed within the ICI cohort to assess agent-specific cardiac outcomes at 1 year. After propensity score matching, the analyses included 4937 matched pairs for nivolumab plus ipilimumab versus pembrolizumab, 3924 matched pairs for nivolumab monotherapy versus nivolumab plus ipilimumab, and 4353 matched pairs for nivolumab monotherapy versus pembrolizumab monotherapy. All outcomes are summarized in Figure 3 and Supplemental Table S3.

#### 3.3.1. Subgroup 1: Nivolumab Plus Ipilimumab Versus Pembrolizumab Monotherapy (*n* = 4937 Matched Pairs)

Combination nivolumab plus ipilimumab was associated with significantly higher rates of composite cardiac conduction disorders compared with pembrolizumab monotherapy (4.5% vs. 2.8%; RR 1.60; 95% CI 1.29–1.99; *p* < 0.001). Both atrioventricular block (2.5% vs. 1.6%; RR 1.53; 95% CI 1.15–2.04; *p* = 0.003) and other conduction disorders (2.7% vs. 1.8%; RR 1.49; 95% CI 1.14–1.95; *p* = 0.003) were significantly elevated. Ventricular tachycardia was also more frequent with combination therapy (1.2% vs. 0.8%; RR 1.58; 95% CI 1.06–2.37; *p* = 0.025), as was heart failure (4.2% vs. 3.3%; RR 1.29; 95% CI 1.05–1.59; *p* = 0.016).

#### 3.3.2. Subgroup 2: Nivolumab Plus Ipilimumab Versus Nivolumab Monotherapy (*n* = 3924 Matched Pairs)

Nivolumab plus ipilimumab was associated with significantly higher rates across all cardiac outcomes compared with nivolumab monotherapy. The composite conduction disorder rate was significantly higher with the combination (4.7% vs. 2.3%; RR 2.05; 95% CI 1.59–2.66; *p* < 0.001), as were atrioventricular block (2.5% vs. 1.2%; RR 2.21; 95% CI 1.55–3.17; *p* < 0.001), other conduction disorders (2.9% vs. 1.5%; RR 1.90; 95% CI 1.39–2.60; *p* < 0.001), ventricular tachycardia (1.4% vs. 0.7%; RR 1.84; 95% CI 1.17–2.87; *p* = 0.007), and heart failure (4.4% vs. 2.9%; RR 1.50; 95% CI 1.19–1.91; *p* = 0.001).

#### 3.3.3. Subgroup 3: Nivolumab Monotherapy Versus Pembrolizumab Monotherapy (*n* = 4353 Matched Pairs)

The two anti-PD-1 monotherapy agents demonstrated broadly similar cardiac outcomes. The composite cardiac conduction disorder rate did not differ significantly (2.6% vs. 3.3%; RR 0.78; 95% CI 0.61–1.01; *p* = 0.059). Atrioventricular block was modestly lower with nivolumab (1.3% vs. 1.9%; RR 0.67; 95% CI 0.48–0.96; *p* = 0.026), while other conduction disorders (1.8% vs. 1.9%; RR 0.93; *p* = 0.667), ventricular tachycardia (0.8% vs. 0.8%; RR 1.00; *p* = 0.992), and heart failure (3.2% vs. 3.8%; RR 0.84; 95% CI 0.67–1.06; *p* = 0.143) were not significantly different.

## 4. Discussion

### 4.1. Summary of Findings

Three principal findings emerged from this analysis. First, among melanoma patients treated with immune checkpoint inhibitors, CTLA-4 exposure (ipilimumab) was associated with a significantly higher incidence of cardiac conduction disorders than anti-PD-1 therapy alone. Second, unlike prior reports suggesting a delayed signal, this excess risk was already present at 1 year and sustained through 3 years, indicating an early and persistent rather than late-emerging toxicity. Third, the risk was graded by lesion severity, culminating in an approximately three-fold excess of complete heart block at 3 years, the most clinically consequential conduction abnormality. Heart failure was also modestly but consistently higher with CTLA-4 exposure at both timepoints.

### 4.2. Within-Class Design and Confounding Control

A central strength of this analysis is its within-class design. Comparisons of immune checkpoint inhibitors against untreated patients or against BRAF/MEK-targeted therapy are confounded by disease stage and by differences in cardiac surveillance intensity, because treatment assignment is tied to tumor genotype and disease burden. By restricting both arms to checkpoint inhibitor recipients and contrasting only CTLA-4 exposure, disease category and surveillance intensity are held approximately constant, so the residual difference in conduction outcomes is more plausibly attributable to CTLA-4 blockade itself. We deliberately did not analyze all-cause mortality as a study outcome, because CTLA-4-containing regimens are preferentially used in higher-stage disease and melanoma stage is not captured in TriNetX; any mortality contrast would reflect this selection rather than cardiac toxicity. Our conclusions therefore rest on conduction-specific endpoints, which are far less susceptible to stage confounding.

### 4.3. CTLA-4-Associated Cardiac Conduction Disorders

The conduction signal localized to CTLA-4 exposure and, notably, was present from the first year (composite RR 1.82 at 1 year) and sustained at 3 years (RR 1.36). The larger relative risks at 1 year argue against a purely late fibrotic mechanism and instead suggest active, CTLA-4-driven immune injury of the conduction system beginning early in the treatment course [19,20]. The most clinically important finding is the roughly three-fold excess of complete heart block at 3 years (RR 3.28), accompanied by a severity gradient in which first- and second-degree block did not differ significantly while complete block was markedly increased. This pattern, escalating effect with escalating lesion severity, is the signature expected from genuine immune-mediated conduction injury rather than incidental detection. The largest pharmacovigilance analysis of ICI cardiac events similarly reported a predilection for CTLA-4-containing regimens [17,18]. Our matched, population-level data extend these observations by quantifying the magnitude and early time course of this risk within an exclusively checkpoint inhibitor-treated population.

### 4.4. Agent-Specific Cardiac Risk: Subgroup Analysis Findings

The three prespecified agent-level subgroup analyses localize the class-level cardiac signal to CTLA-4 (ipilimumab) exposure. Combination nivolumab plus ipilimumab carried significantly higher rates of conduction disorders, ventricular tachycardia, and heart failure than pembrolizumab monotherapy, whereas the two anti-PD-1 monotherapies had broadly equivalent cardiac profiles. These findings indicate that patients requiring anti-PD-1 monotherapy carry a lower inherent cardiac conduction risk, and that heightened surveillance should be prioritized for those receiving or having received anti-CTLA-4-containing regimens. Because the CTLA-4-exposed cohort combined ipilimumab monotherapy with nivolumab–ipilimumab combination therapy, these subgroup analyses are central to interpreting the signal: adding ipilimumab to nivolumab sharply increased conduction risk, whereas the two anti-PD-1 monotherapies were low and statistically indistinguishable, indicating that the excess toxicity tracks with CTLA-4 (ipilimumab) exposure rather than with PD-1 blockade. We nonetheless could not isolate ipilimumab monotherapy as a separate arm, so an additional contribution from dual checkpoint blockade cannot be fully excluded. Anti-PD-1 therapy is nonetheless not free of conduction risk, and its mechanism warrants comment. PD-1 and its ligand PD-L1 are expressed in cardiomyocytes and the cardiac conduction system, where the PD-1/PD-L1 axis acts as a local cardioprotective checkpoint that restrains T-cell–mediated myocardial inflammation; pharmacologic PD-1 blockade can remove this brake and permit lymphocytic infiltration of the myocardium and atrioventricular node, and PD-1-monotherapy myocarditis and conduction disease, although less frequent than with CTLA-4-containing regimens, are well documented. This provides a plausible basis for the low but non-zero conduction event rates seen in both anti-PD-1 arms. Between the two anti-PD-1 agents, atrioventricular block was modestly less frequent with nivolumab than with pembrolizumab (RR 0.67; 95% CI 0.48–0.96; *p* = 0.026), the only statistically significant between-agent difference, which we interpret cautiously as hypothesis-generating and potentially related to differences in dosing schedule, cumulative exposure, or residual unmeasured population characteristics rather than a definite pharmacologic distinction.

### 4.5. Heart Failure and Ventricular Arrhythmias

Heart failure was modestly but significantly higher with CTLA-4 exposure at both timepoints (3-year RR 1.17; 1-year RR 1.35), consistent with the recognized association between dual checkpoint blockade and immune-mediated myocardial injury [13,14]. Ventricular tachycardia showed only a borderline, non-significant signal that did not persist (1-year RR 1.42, *p* = 0.061; 3-year RR 1.13, *p* = 0.401), so any contribution of CTLA-4 exposure to ventricular arrhythmia remains uncertain and warrants prospective evaluation [21]. Because both arms received immune checkpoint inhibitors, these differences are unlikely to reflect checkpoint-inhibitor exposure per se and instead point to an incremental contribution of CTLA-4 blockade.

### 4.6. Clinical Implications

These findings support several practice recommendations. First, because the excess conduction risk with CTLA-4 exposure is present from the first year, electrocardiographic surveillance should begin during treatment rather than only after its completion; our findings support consideration of a baseline ECG before initiation of CTLA-4-containing therapy, monitoring during treatment, and periodic ECG surveillance during subsequent follow-up [22,23,24]. Second, the three-fold excess of complete heart block identifies CTLA-4-containing regimens as the highest-risk group for clinically serious conduction disease, warranting heightened vigilance and a low threshold for cardiology referral. Third, new palpitations, presyncope, syncope, or exertional dyspnea in any checkpoint inhibitor-treated patient should trigger urgent ECG evaluation. Fourth, multidisciplinary cardio-oncology collaboration is essential to ensure that cardiac concerns do not lead to inappropriate discontinuation of effective immunotherapy [23,25,26,27].

### 4.7. Strengths and Limitations

This analysis compared more than 18,000 immune checkpoint inhibitor-treated melanoma patients across 111 US healthcare organizations, with 6841 matched pairs providing power to detect differences in uncommon conduction outcomes. By restricting both arms to checkpoint inhibitor recipients and contrasting only CTLA-4 exposure, the design holds disease category and cardiac-surveillance intensity approximately constant, addressing two major sources of confounding that affect comparisons against untreated or targeted-therapy populations. Propensity score matching achieved excellent balance (all SMDs <0.03), and the real-world population enhances generalizability beyond trial-selected patients.

Several limitations warrant consideration. The retrospective design precludes causal inference. Although both arms received checkpoint inhibitors, residual confounding by indication remains, because CTLA-4-containing regimens are preferentially used in higher-stage, more aggressive disease; melanoma stage, treatment line, and treatment duration are unavailable in TriNetX. To quantify how much unmeasured confounding would be required to explain the primary result, we calculated an E-value for the 1-year composite outcome: an unmeasured confounder would need to be associated with both CTLA-4 exposure and cardiac conduction disorders by a risk ratio of at least 3.04 each, beyond the covariates already balanced by matching, to reduce the observed association (risk ratio 1.82) to the null, and by at least 2.32 to move the lower confidence bound to 1.0. Because disease stage, although unrecorded, is unlikely to exert an independent effect of this magnitude on the conduction system beyond the balanced cardiovascular comorbidities, residual confounding is unlikely to account for the entire signal, although it cannot be excluded. Smoking status, alcohol use, and socioeconomic or insurance-related access to care were likewise not captured and could not be entered into the propensity model, despite their potential to influence both regimen selection and baseline cardiovascular risk. Administrative coding may underestimate conduction abnormalities that do not prompt a formal diagnosis, and ICD-10 coding does not permit reliable subclassification within categories (for example, Mobitz I versus II second-degree block). In addition, although restricting both arms to checkpoint-inhibitor recipients reduces, but does not eliminate, differential surveillance, patients receiving combination therapy may undergo more intensive cardiac monitoring and electrocardiographic testing, which could increase the ascertainment of conduction abnormalities and partly inflate the observed association. This concern applies chiefly to the milder, frequently asymptomatic abnormalities, such as first-degree atrioventricular block and other minor conduction delays, which are detected only when an electrocardiogram is obtained and would therefore be captured preferentially under more intensive monitoring. It applies far less to complete heart block, which is typically symptomatic and comes to clinical attention regardless of surveillance intensity. That the strongest and most severe signal in our data, the roughly three-fold excess of complete heart block, is also the outcome least susceptible to differential ascertainment argues that this association reflects a genuine biological effect rather than detection bias alone. Complete heart block was assessed at 3 years only. Moreover, although the relative risk for complete heart block was large, it rested on a relatively small number of absolute events (36 versus 11 cases), as did the estimates for other rare conduction subtypes; these associations should therefore be interpreted with corresponding caution and warrant confirmation in larger cohorts. A further constraint is that the TriNetX platform compares only two cohorts at a time and does not support simultaneous multi-group analysis; the three regimen subgroups could therefore be evaluated only through separate, individually matched pairwise comparisons rather than a single omnibus test. Finally, the primary composite spans conduction abnormalities of widely differing clinical significance, from asymptomatic bundle-branch block to complete heart block; although the severity-stratified analysis partly mitigates this, the heterogeneity of the composite is itself a limitation, distinct from the administrative-coding granularity noted above. Finally, the rarer conduction subtypes were underpowered at 1 year, so non-significant early findings for these specific lesions should be interpreted as inconclusive rather than negative.

## 5. Conclusions

In this large, multicenter, propensity score-matched analysis of immune checkpoint inhibitor-treated melanoma patients, CTLA-4 exposure (ipilimumab) was associated with a significantly higher incidence of cardiac conduction disorders than anti–PD-1 therapy alone. This excess was present within the first year and sustained at 3 years, followed a severity gradient, and culminated in an approximately three-fold increase in complete heart block. Heart failure was also modestly higher with CTLA-4 exposure at both timepoints. Because the CTLA-4-exposed cohort included both ipilimumab monotherapy and nivolumab plus ipilimumab and the agent-level analyses localized the excess to the combination regimen, this risk should be attributed to CTLA-4-containing (predominantly combination) therapy rather than to ipilimumab monotherapy alone.

These findings identify CTLA-4-containing immunotherapy as carrying a distinct, early, and severity-graded risk of conduction system disease. They support incorporating electrocardiographic surveillance into treatment from initiation onward, rather than only after therapy, for patients receiving CTLA-4-containing regimens, while preserving the substantial oncologic benefits of immune checkpoint inhibition.

## Figures and Tables

**Table 1:** Baseline Characteristics Before and After Propensity Score Matching.

Variable	Before Matching (*n* = 7313 vs. 11,481)				After Matching (*n* = 6841 vs. 6841)			
	Ipilimumab	Anti–PD-1	*p*	SMD	Ipilimumab	Anti–PD-1	*p*	SMD
**Demographics**								
Age at index, years (mean ± SD)	61.8 ± 14.0	66.8 ± 14.2	<0.001	0.353	62.9 ± 13.3	62.6 ± 14.6	0.220	0.021
Current age, years (mean ± SD)	67.3 ± 14.0	71.3 ± 13.9	<0.001	0.286	68.2 ± 13.5	67.8 ± 14.6	0.099	0.028
Female sex	2765 (37.9%)	4389 (38.3%)	0.556	0.009	2596 (37.9%)	2584 (37.8%)	0.832	0.004
White	6424 (88.1%)	10,003 (87.4%)	0.156	0.021	6012 (87.9%)	5972 (87.3%)	0.300	0.018
Black or African American	99 (1.4%)	141 (1.2%)	0.456	0.011	93 (1.4%)	95 (1.4%)	0.883	0.003
Hispanic or Latino	213 (2.9%)	221 (1.9%)	<0.001	0.064	168 (2.5%)	181 (2.6%)	0.481	0.012
Asian	89 (1.2%)	169 (1.5%)	0.143	0.022	87 (1.3%)	94 (1.4%)	0.600	0.009
**Comorbidities**								
Hypertension	3124 (42.8%)	5630 (49.2%)	<0.001	0.128	3006 (43.9%)	2940 (43.0%)	0.255	0.019
Diabetes mellitus	1155 (15.8%)	2074 (18.1%)	<0.001	0.061	1123 (16.4%)	1063 (15.5%)	0.162	0.024
Hyperlipidemia	1942 (26.6%)	3884 (33.9%)	<0.001	0.159	1899 (27.8%)	1871 (27.3%)	0.592	0.009
Chronic kidney disease	466 (6.4%)	1158 (10.1%)	<0.001	0.136	459 (6.7%)	440 (6.4%)	0.512	0.011
Coronary artery disease	901 (12.4%)	1977 (17.3%)	<0.001	0.139	891 (13.0%)	853 (12.5%)	0.330	0.017
Heart failure	319 (4.4%)	868 (7.6%)	<0.001	0.136	317 (4.6%)	307 (4.5%)	0.682	0.007
Atrial fibrillation/flutter	565 (7.7%)	1341 (11.7%)	<0.001	0.134	558 (8.2%)	528 (7.7%)	0.343	0.016
Obesity	1289 (17.7%)	2134 (18.6%)	0.095	0.025	1234 (18.0%)	1186 (17.3%)	0.282	0.018
Cerebral infarction	231 (3.2%)	463 (4.0%)	0.002	0.047	222 (3.2%)	193 (2.8%)	0.148	0.025
Other malignancy	1198 (16.4%)	1250 (10.9%)	<0.001	0.161	978 (14.3%)	965 (14.1%)	0.750	0.005
**Baseline antiarrhythmic/rate-control medications**								
Beta-blockers	2570 (35.2%)	4395 (38.4%)	<0.001	0.065	2426 (35.5%)	2378 (34.8%)	0.390	0.015
Amiodarone	131 (1.8%)	304 (2.7%)	<0.001	0.058	128 (1.9%)	127 (1.9%)	0.950	0.001
Sotalol	34 (0.5%)	60 (0.5%)	0.584	0.008	34 (0.5%)	33 (0.5%)	0.903	0.002
Flecainide	41 (0.6%)	67 (0.6%)	0.838	0.003	41 (0.6%)	40 (0.6%)	0.911	0.002
Dofetilide	13 (0.2%)	20 (0.2%)	0.955	0.001	12 (0.2%)	14 (0.2%)	0.695	0.007
Propafenone	24 (0.3%)	38 (0.3%)	0.973	0.001	24 (0.4%)	23 (0.3%)	0.884	0.002
Dronedarone	15 (0.2%)	24 (0.2%)	0.953	0.001	14 (0.2%)	13 (0.2%)	0.847	0.003

Values are *n* (%) or mean ± SD. SMD = standardized mean difference; PD-1 = programmed cell death protein 1.

**Table 2:** Clinical Outcomes at 1 Year and 3 Years After Propensity Score Matching.

Outcome	Ipilimumab (CTLA-4)	Anti–PD-1	Risk Ratio (95% CI)	*p*-Value
**1-Year Outcomes**				
Cardiac conduction disorder composite	3.8% (244/6466)	2.1% (134/6465)	1.82 (1.48–2.24)	<0.001
Atrioventricular block (I44)	2.3% (149/6617)	1.1% (71/6619)	2.10 (1.59–2.78)	<0.001
Other conduction disorders (I45)	2.2% (144/6631)	1.4% (94/6633)	1.53 (1.18–1.98)	0.001
First-degree AV block (I44.0)	0.8% (53/6737)	0.5% (32/6746)	1.66 (1.07–2.57)	0.022
Second-degree AV block (I44.1)	0.2% (15/6816)	0.2% (12/6820)	1.25 (0.59–2.67)	0.562
Ventricular tachycardia (I47.2)	1.0% (68/6752)	0.7% (48/6760)	1.42 (0.98–2.05)	0.061
Heart failure (I50)	4.0% (259/6523)	2.9% (192/6527)	1.35 (1.12–1.62)	0.001
**3-Year Outcomes**				
Cardiac conduction disorder composite	5.1% (328/6466)	3.7% (241/6465)	1.36 (1.16–1.60)	<0.001
Atrioventricular block (I44)	3.0% (198/6617)	2.0% (133/6619)	1.49 (1.20–1.85)	<0.001
Other conduction disorders (I45)	3.0% (202/6631)	2.4% (161/6633)	1.26 (1.02–1.54)	0.029
First-degree AV block (I44.0)	1.1% (74/6737)	1.0% (67/6746)	1.11 (0.80–1.54)	0.548
Second-degree AV block (I44.1)	0.3% (22/6816)	0.2% (15/6820)	1.47 (0.76–2.83)	0.248
Complete heart block (I44.2)	0.5% (36/6807)	0.2% (11/6812)	3.28 (1.67–6.43)	<0.001
Ventricular tachycardia (I47.2)	1.4% (94/6752)	1.2% (83/6760)	1.13 (0.85–1.52)	0.401
Heart failure (I50)	5.7% (375/6523)	4.9% (322/6527)	1.17 (1.01–1.35)	0.038

Risk ratios >1.0 indicate higher event rates in the CTLA-4-exposed (ipilimumab) cohort. Percentages are the cumulative incidence within each follow-up window. Complete heart block was reportable at 3 years only; at 1 year the number of events fell below the TriNetX minimum cell-count threshold and was suppressed rather than analyzable. CTLA-4 = cytotoxic T-lymphocyte-associated antigen 4; PD-1 = programmed cell death protein 1.

## Data Availability

The data analyzed in this study were obtained from the TriNetX Research Network (https://trinetx.com, accessed on 9 August 2026) and are available to credentialed researchers under a data-use agreement. The authors are not permitted to redistribute the underlying patient-level data; the aggregate results supporting the findings of this study are available from the corresponding author upon reasonable request.

## Supplementary Materials

The following supporting information can be downloaded at https://www.mdpi.com/article/10.3390/cancers18162608/s1, Table S1. Clinical Outcomes at 1 Year and 3 Years Before Propensity Score Matching; Table S2. Baseline Characteristics Before Propensity Score Matching; Table S3. ICI Agent Subgroup Analysis: Cardiac Outcomes at 1 Year After Propensity Score Matching.
