# Supplementary material for: Cardiac Conduction Disorders in Melanoma Patients Treated with CTLA-4-Containing Versus PD-1-Only Immune Checkpoint Inhibitor Therapy: A Propensity Score-Matched Real-World Analysis
Source: Cancers (Basel). 2026 Aug 13;18(16):2608. doi: 10.3390/cancers18162608 (PMC13511609; doi:10.3390/cancers18162608)
Supplement: Supplementary file 1 [file cancers-18-02608-s001.zip › cancers-4412112-supplementary.pdf]

Supplementary Materials

Table S1. Clinical Outcomes at 1 Year and 3 Years Before Propensity Score Matching. *Unmatched cohorts (ipilimumab, n = 7,313; anti–PD-1, n = 11,481). Risk ratios > 1 indicate higher risk with ipilimumab. P-values are from the associated risk-difference test. Denominators vary by outcome because patients with the outcome before the time window were excluded.*

Outcome (ICD-10-CM)	Ipilimumab, n (%)	Anti–PD-1, n (%)	Risk Ratio (95% CI)	P
**1 Year**				
Composite conduction disorder	255 (3.7%)	301 (2.8%)	1.298 (1.101–1.529)	0.002
Atrioventricular block (I44)	154 (2.2%)	173 (1.6%)	1.374 (1.108–1.704)	0.004
Other conduction disorders (I45)	151 (2.1%)	197 (1.8%)	1.191 (0.965–1.469)	0.103
First-degree AV block (I44.0)	55 (0.8%)	81 (0.7%)	1.059 (0.753–1.489)	0.743
Second-degree AV block (I44.1)	15 (0.2%)	25 (0.2%)	0.941 (0.496–1.783)	0.851
Ventricular tachycardia (I47.2)	71 (1.0%)	84 (0.7%)	1.324 (0.966–1.813)	0.080
Heart failure (I50)	270 (3.9%)	374 (3.5%)	1.094 (0.939–1.276)	0.249
**3 Years**				
Composite conduction disorder	344 (5.0%)	481 (4.5%)	1.096 (0.957–1.254)	0.185
Atrioventricular block (I44)	204 (2.9%)	281 (2.6%)	1.121 (0.938–1.339)	0.208
Other conduction disorders (I45)	213 (3.0%)	319 (2.9%)	1.037 (0.874–1.230)	0.676
First-degree AV block (I44.0)	77 (1.1%)	141 (1.3%)	0.852 (0.646–1.122)	0.253
Second-degree AV block (I44.1)	22 (0.3%)	31 (0.3%)	1.113 (0.645–1.920)	0.702
Complete heart block (I44.2)	37 (0.5%)	33 (0.3%)	1.756 (1.099–2.805)	0.017
Ventricular tachycardia (I47.2)	99 (1.4%)	133 (1.2%)	1.166 (0.900–1.510)	0.244
Heart failure (I50)	396 (5.7%)	601 (5.7%)	0.999 (0.883–1.130)	0.986

AV, atrioventricular; CI, confidence interval. Matched results are shown in Table 2 of the main text.

Table S2. Baseline Characteristics Before Propensity Score Matching. *Unmatched cohorts. P-values compare ipilimumab versus anti–PD-1; SMD values > 0.10 indicate meaningful imbalance. The same characteristics after matching (all SMD < 0.03) are shown in Table 1 of the main text.*

Variable	Ipilimumab (n = 7,313)	Anti–PD-1 (n = 11,481)	P	SMD
**Demographics**				
Age at index, years (mean ± SD)	61.8 ± 14.0	66.8 ± 14.2	<0.001	0.353
Current age, years (mean ± SD)	67.3 ± 14.0	71.3 ± 13.9	<0.001	0.286
Female sex	2,765 (37.9%)	4,389 (38.3%)	0.556	0.009
White	6,424 (88.1%)	10,003 (87.4%)	0.156	0.021
Black or African American	99 (1.4%)	141 (1.2%)	0.456	0.011
Hispanic or Latino	213 (2.9%)	221 (1.9%)	<0.001	0.064
Asian	89 (1.2%)	169 (1.5%)	0.143	0.022
**Comorbidities**				

Variable	Ipilimumab (n = 7,313)	Anti–PD-1 (n = 11,481)	P	SMD
Hypertension	3,124 (42.8%)	5,630 (49.2%)	<0.001	0.128
Diabetes mellitus	1,155 (15.8%)	2,074 (18.1%)	<0.001	0.061
Hyperlipidemia	1,942 (26.6%)	3,884 (33.9%)	<0.001	0.159
Chronic kidney disease	466 (6.4%)	1,158 (10.1%)	<0.001	0.136
Coronary artery disease	901 (12.4%)	1,977 (17.3%)	<0.001	0.139
Heart failure	319 (4.4%)	868 (7.6%)	<0.001	0.136
Atrial fibrillation/flutter	565 (7.7%)	1,341 (11.7%)	<0.001	0.134
Obesity	1,289 (17.7%)	2,134 (18.6%)	0.095	0.025
Cerebral infarction	231 (3.2%)	463 (4.0%)	0.002	0.047
Other malignancy	1,198 (16.4%)	1,250 (10.9%)	<0.001	0.161
**Baseline antiarrhythmic / rate-control medications**				
Beta-blockers	2,570 (35.2%)	4,395 (38.4%)	<0.001	0.065
Amiodarone	131 (1.8%)	304 (2.7%)	<0.001	0.058
Sotalol	34 (0.5%)	60 (0.5%)	0.584	0.008
Flecainide	41 (0.6%)	67 (0.6%)	0.838	0.003
Dofetilide	13 (0.2%)	20 (0.2%)	0.955	0.001
Propafenone	24 (0.3%)	38 (0.3%)	0.973	0.001
Dronedarone	15 (0.2%)	24 (0.2%)	0.953	0.001

SD, standard deviation; SMD, standardized mean difference.

Table S3. ICI Agent Subgroup Analysis: Cardiac Outcomes at 1 Year After Propensity Score Matching. *Three prespecified pairwise comparisons within the immune checkpoint inhibitor cohort, each propensity score–matched 1:1. A risk ratio > 1 indicates higher risk in Group 1; < 1 indicates lower risk in Group 1.*

Outcome	Group 1, %	Group 2, %	Risk Ratio (95% CI)	P
**Nivolumab + ipilimumab (Group 1; 4,937 pairs) vs. pembrolizumab monotherapy (Group 2)**				
Composite conduction disorder	4.5%	2.8%	1.603 (1.291–1.989)	<0.001
Atrioventricular block	2.5%	1.6%	1.532 (1.153–2.036)	0.003
Other conduction disorders	2.7%	1.8%	1.493 (1.141–1.952)	0.003
Ventricular tachycardia	1.2%	0.8%	1.583 (1.057–2.372)	0.025
Heart failure	4.2%	3.3%	1.290 (1.049–1.586)	0.016
**Nivolumab monotherapy (Group 1; 3,924 pairs) vs. nivolumab + ipilimumab (Group 2)**				
Composite conduction disorder	2.3%	4.7%	0.487 (0.376–0.630)	<0.001
Atrioventricular block	1.2%	2.5%	0.452 (0.316–0.645)	<0.001
Other conduction disorders	1.5%	2.9%	0.526 (0.384–0.721)	<0.001

Outcome	Group 1, %	Group 2, %	Risk Ratio (95% CI)	P
Ventricular tachycardia	0.7%	1.4%	0.545 (0.348–0.856)	0.007
Heart failure	2.9%	4.4%	0.665 (0.523–0.844)	0.001
**Nivolumab monotherapy (Group 1; 4,353 pairs) vs. pembrolizumab monotherapy (Group 2)**				
Composite conduction disorder	2.6%	3.3%	0.782 (0.606–1.010)	0.059
Atrioventricular block	1.3%	1.9%	0.674 (0.475–0.956)	0.026
Other conduction disorders	1.8%	1.9%	0.933 (NR)	0.667
Ventricular tachycardia	0.8%	0.8%	0.998 (NR)	0.992
Heart failure	3.2%	3.8%	0.843 (0.670–1.060)	0.143

CI, confidence interval; NR, confidence interval not reported for non-significant secondary outcomes in the source output. Corresponds to Figure 3 of the main text.
